# Comprehensive Overview of the Brassinosteroid Biosynthesis Pathways: Substrates, Products, Inhibitors, and Connections

**DOI:** 10.3389/fpls.2020.01034

**Published:** 2020-07-07

**Authors:** Andrzej Bajguz, Magdalena Chmur, Damian Gruszka

**Affiliations:** ^1^ Faculty of Biology, University of Bialystok, Bialystok, Poland; ^2^ Faculty of Natural Sciences, Institute of Biology, Biotechnology and Environmental Protection, University of Silesia, Katowice, Poland

**Keywords:** brassinazole, brassinolide, castasterone, inhibitors, mevalonate and nonmevalonate pathways, sterols

## Abstract

Brassinosteroids (BRs) as a class of steroid plant hormones participate in the regulation of numerous developmental processes, including root and shoot growth, vascular differentiation, fertility, flowering, and seed germination, as well as in responding to environmental stresses. During four decades of research, the BR biosynthetic pathways have been well studied with forward- and reverse genetics approaches. The free BRs contain 27, 28, and 29 carbons within their skeletal structure: (1): 5α-cholestane or 26-nor-24α-methyl-5α-cholestane for C_27_-BRs; (2) 24α-methyl-5α-cholestane, 24β-methyl-5α-cholestane or 24-methylene-5α-cholestane for C_28_-BRs; (3) 24α-ethyl-5α-cholestane, 24(*Z*)-ethylidene-5α-cholestane, 25-methyl-5α-campestane or 24-methylene-25-methyl-5α-cholestane for C_29_-BRs, as well as different kinds and orientations of oxygenated functions in A- and B-ring. These alkyl substituents are also common structural features of sterols. BRs are derived from sterols carrying the same side chain. The C_27_-BRs without substituent at C-24 are biosynthesized from cholesterol. The C_28_-BRs carrying either an α-methyl, β-methyl, or methylene group are derived from campesterol, 24-epicampesterol or 24-methylenecholesterol, respectively. The C_29_-BRs with an α-ethyl group are produced from sitosterol. Furthermore, the C_29_ BRs carrying methylene at C-24 and an additional methyl group at C-25 are derived from 24-methylene-25-methylcholesterol. Generally, BRs are biosynthesized *via* cycloartenol and cycloartanol dependent pathways. Till now, more than 17 compounds were characterized as inhibitors of the BR biosynthesis. For nine of the inhibitors (e.g., brassinazole and YCZ-18) a specific target reaction within the BR biosynthetic pathway has been identified. Therefore, the review highlights comprehensively recent advances in our understanding of the BR biosynthesis, sterol precursors, and dependencies between the C_27_-C_28_ and C_28_-C_29_ pathways.

## Introduction

Brassinosteroids (BRs) represent the sixth class of plant hormones. Since the discovery of brassinolide (BL) in 1979, about 70 naturally occurring compounds from this group have been reported as free molecules or conjugates with glucose and fatty acids. BRs are structurally very similar to androgens, estrogens, corticoids, and ecdysteroids. Their presence was reported both in lower and higher plants, especially in angiosperms; and also in all plant organs, including roots, stems, leaves, flowers, anthers, pollen, seeds, and grain (Bajguz and Tretyn, 2003; Yokota et al., 2017; Bajguz, 2019; Zullo and Bajguz, 2019). BRs play an essential role in the development and growth of plants. They elicit a broad spectrum of morphological and physiological responses as well as a tolerance against abiotic and biotic stress (Bajguz and Hayat, 2009; Bajguz and Piotrowska-Niczyporuk, 2014; Wei and Li, 2016; Wendeborn et al., 2017; Ahanger et al., 2018; Siddiqui et al., 2018; Nolan et al., 2020).

## Chemical Structure of BRs

Based on the total number of carbons, BRs are divided into C_27_, C_28_, and C_29_-type. The basic structure of C_27_-BRs is a 5α-cholestane skeleton, C_28_-BRs: 5α-ergostane, and C_29_-BRs: 5α-stigmastane (**Figure S1**). Differences in the structure of these hormones are due to the type and orientation of oxygenated functions in the A- and B-ring, as well as the number and position of functional groups in the side chain of the molecule. These modifications arise during oxidation and reduction reactions. Based on the cholesterol (CR) side chain, BRs are divided by different substituents into C-23, C-24, C-25, 23-oxo, 24*S*-methyl, 24*R*-methyl, 24-methylene, 24*S*-ethyl, 24-ethylidene, 24-methylene-25-methyl, 24-methyl-25-methyl; without substituent at C-23, without substituent at C-24, and without substituents at C-23, C-24. BRs can also conjugate with glucose and fatty acids (Fujioka and Yokota, 2003; Bajguz, 2007; Piotrowska and Bajguz, 2011; Wendeborn et al., 2017; Zullo and Bajguz, 2019).

## BRs Biosynthetic Pathways

**Figure 1:**
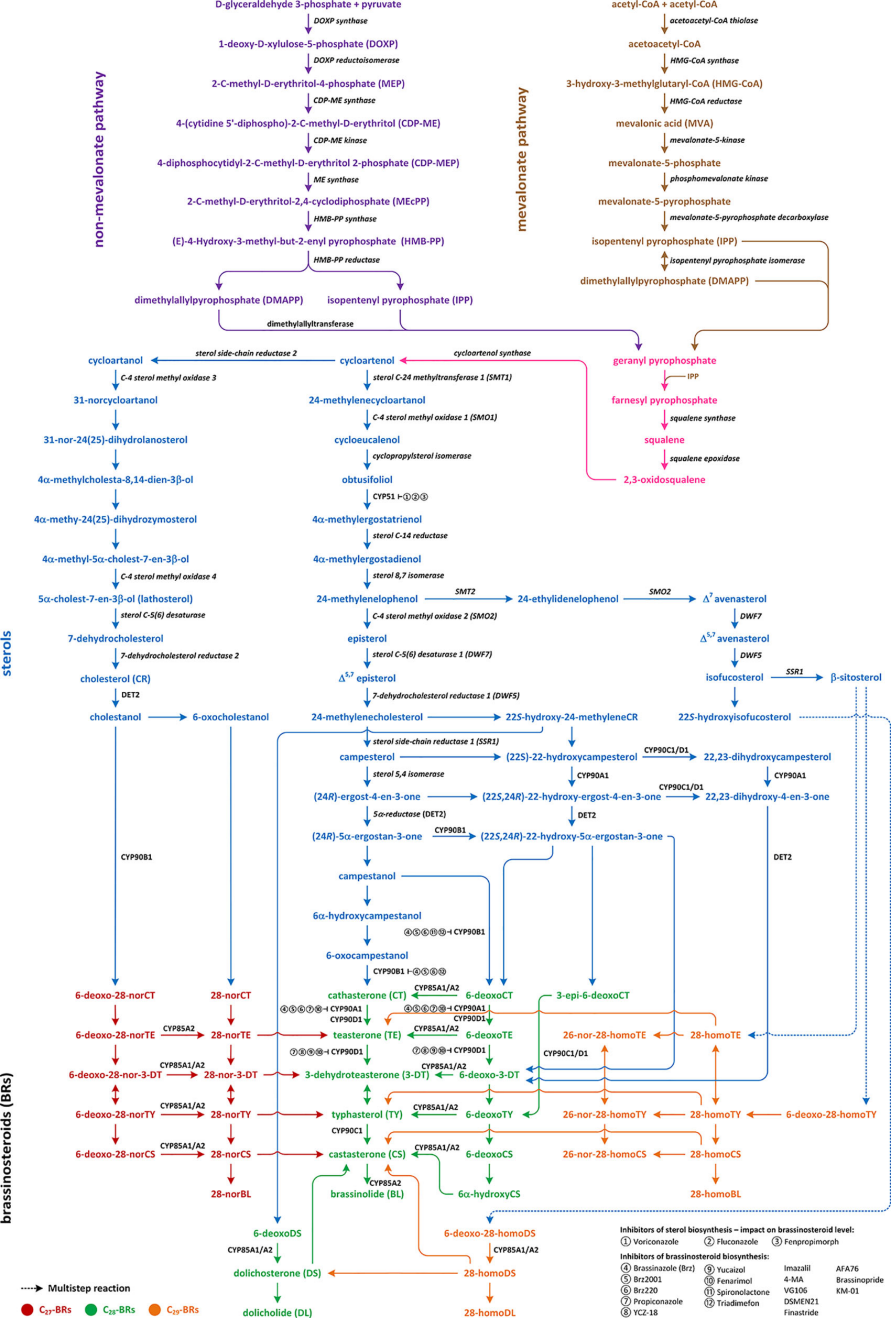
Multistep reactions of brassinosteroids biosynthesis and their sterol biosynthetic precursors.

Three pathways of the BR biosynthesis leading to the production of C_27_-, C_28_-, or C_29_-type of BRs are currently known (**Figure 1**; **Figure S1**). Early steps of their synthesis are common for each type and may occur *via* mevalonate (MVA) or non-MVA pathway, while later steps differentiate the BR biosynthesis pathways (cycloartenol- and cycloartanol-dependent). So far, most of the reactions, enzymes, and genes were discovered and characterized by the C_28_-BR biosynthesis pathway (mostly in *Arabidopsis thaliana*, from which the majority of genes in this pathway were isolated). Their biosynthesis includes two major stages: biosynthesis of campesterol and 22α-hydroxycampesterol. The direct substrate of C_27_-BRs viz. cholesterol (CR) is finally converted to 28-norBL, whereas the biosynthesis of C_29_-BRs is initiated from β-sitosterol and leads to 28-homoBL. However, not all indirect compounds of these two pathways have been identified (**Figure 1**; **Figure S1**) (Fujioka et al., 2002; Kwon and Choe, 2005; Fujita et al., 2006; Ohnishi et al., 2006b; Chung and Choe, 2013; Roh et al., 2017; Kim et al., 2018; Rozhon et al., 2019).

### Early Steps of BRs Biosynthesis

Biosynthesis of isopentenyl pyrophosphate (IPP), which is an indirect compound in the CR synthesis pathway, can occur *via* two pathways: non-MVA in lower plants and MVA in the most of higher plants. The initial compounds in non-MVA pathway are D-glyceraldehyde 3-phosphate and pyruvate, which are transformed into the 1-deoxy-D-xylulose 5-phosphate (DOXP) by the DOXP synthase. Then, DOXP is converted to 2-C-methyl-D-erythritol 4-phosphate (MEP) by the DOXP reductoisomerase. The next step leads to the formation of 4-(cytidine 5’-diphospho)-2-C methyl-D-erythritol (CDP-ME) from MEP. The reaction is catalyzed by the CDP-ME synthase. Then, CDP-ME is converted to CDP-methyl-D-erythritol-2-phosphate by the CDP-ME kinase, and the obtained compound is transformed to 2-C-methyl-D-erythitol-2,4-cyclodiphosphate by the ME cyclodiphosphate synthase. Finally, with the action of reductases and dehydratases, the IPP is synthesized (**Figure 1**; **Figure S1**) (Lichtenthaler, 2000). In the MVA pathway, three molecules of acetyl-CoA are combined to produce 3-hydroxy-3-methyloglutaryl-CoA by the HMG-CoA synthase. The obtained compound is reduced to MVA by the HMG-CoA reductase. IPP is synthesized from MVA through the two indirect phosphorylation intermediates, such as MVA-phosphate and MVA-pyrophosphate (MVA-PP) (Miziorko, 2011). Enzymes involved in these reactions are the MVA kinase, phospho-MVA kinase, and MVA-PP decarboxylase, respectively (**Figure 1**; **Figure S1**) (Wang et al., 2017).

Biochemical changes of IPP *via* geranyl pyrophosphate and farnesyl pyrophosphate lead to the synthesis of squalene, which is oxidized to squalene-2,3-oxide *via* squalene epoxidase and the latter is converted to cycloartenol by the cycloartenol synthase. Cycloartenol is a key compound for the BR biosynthesis because it constitutes a substrate for multistep reactions in few pathways, which lead to the synthesis of C_27_-, C_28_-, and C_29_-BRs. Conversion of cycloartenol *via* cycloartanol and in a series of subsequent reactions to cholesterol/cholestanol and/or 6-oxocholestanol leads to the synthesis of C_27_-BRs (**Figure 1**; **Figure S1**) (Wang et al., 2017).

Cycloartenol may also be a substrate of the C-24 methylation reaction, which is catalyzed by the sterol C-24 methyltransferase (SMT1), and leads to 24-methylenecycloartanol. The next few reactions are catalyzed by C-4 sterol methyl oxidase (SMO1), cycloprophylsterol isomerase, obtusifoliol 14α-demethylase (CYP51), and sterol C-14 reductase, leading to the synthesis of 4α-methylergostatrienol. Indirect products of these reactions are cycloeucalenol and obtusifoliol. In next step, the sterol C-14 reductase which is encoded by the *FACKEL*/*HYDRA2* gene catalyzes the reduction of 4α-methylergostatrienol to 4α-methylergostadienol, which is converted in the subsequent reaction to 24-methylenelophenol by the sterol 8,7 isomerase (Kushiro et al., 2001; Schneider, 2002; Sonawane et al., 2016; Wang et al., 2017).

24-methylenelophenol is a substrate of two independent pathways of sterol biosynthesis. The first leads to the biosynthesis of isofucosterol/β-sitosterol that are precursors of the C_29_-BR biosynthesis (Xin et al., 2016); the second pathway, 24-converts methylenelophenol to campesterol, which is a substrate of the C_28_-BR biosynthesis (**Figure 1**; **Figure S1**) (Choe, 2006; Sonawane et al., 2016).

### Biosynthesis of C_27_-BRs

The C_27_-BR biosynthesis pathway starts from the conversion of cycloartenol to cycloartanol (**Figure 1**; **Figure S1**) by the sterol side chain reductase 2 (SSR2) and proceeds through a synthesis of 31-norcycloartanol from cycloartanol by the C4-sterol methyloxidase3 (SMO3), and further biochemical changes of 31-norcycloartanol up to 31-nor-24(25)-dihydrolanosterol, 4α-methylcholestadienol, 4α-methyl-24(25)-dihydrozymosterol, 4α-methylcholestenol, 5α-cholest-7-en-3β-ol, 7-dehydroCR and finally CR, respectively. The reaction of 5α-cholest-7-en-3β-ol synthesis is catalyzed by the C4-sterol methyloxidase 4 (SMO4), while sterol C5(6) desaturase catalyzes synthesis of 7-dehydrocholesterol. Cholesterol is synthesized from 7-dehydrocholesterol by the 7-dehydrocholesterol reductase 2 (Sonawane et al., 2016). The biosynthesis of C_27_-BRs might occur through the late C6 oxidation pathway. Firstly, cholesterol is converted to cholestanol (a C_27_-BR biosynthesis precursor) by the 5α-reductase encoded by the *DET2* gene. The 5α-reductase DET2 belongs to enzymes that have broad substrate specificity; therefore, it catalyzes reaction (**Figure 1**; **Figure S1**). In the next steps of the C_27_-BR biosynthesis pathway 6-deoxo-28-norcathasterone, 6-deoxo-28-norteasterone, 6-deoxo-28-nor-3-dehydroteasterone, 6-deoxo-28-nortyphasterol and 6-deoxo-28-norcastasterone are synthesized in the consecutive reactions. Furthermore, the early C6 oxidation pathway is initiated through oxidation of cholestanol into 6-oxocholestanol, which is then followed by a synthesis of 28-norcathasterone, 28-norteasterone, 28-nor-3-dehydroteasterone, 28-nortyphasterol, 28-norcastasterone, and 28-norbrassinolide. Enzymatic conversions of compounds from the late C6 oxidation pathway to the early C6 oxidation counterparts have been evidenced, e.g. 6-deoxo-28-norteasterone to 28-norteasterone, 6-deoxo-28-nortyphasterol to 28-nortyphasterol, and 6-deoxo-28-norcastasterone to 28-norcastasterone. It is known that oxidation/hydroxylation steps in the all BR biosynthetic pathways are catalyzed by cytochrome P450 enzymes. The CYP85A1 and CYP85A2 oxidases, similarly to the 5α-reductase DET2, belong to enzymes of broad substrate specificity. They catalyze the oxidation reactions connecting the late and early counterparts of the C_27_-BR biosynthesis pathway, and also catalyze corresponding reactions during the C_28_-BR pathway (**Figure 1**; **Figure S1**) (Kim et al., 2005; Fujita et al., 2006; Joo et al., 2012; Zhao and Li, 2012; Joo et al., 2015).

### Biosynthesis of C_28_-BRs

24-methylenelophenol may also be converted to episterol, which is the first characteristic metabolite in the C_28_-BR biosynthesis. The reaction is catalyzed by the C-4 sterol methyl oxidase 2 (SMO2) (**Figure 1**; **Figure S1**). Then, episterol is converted to 5-dehydroepisterol by the sterol C-5(6) desaturase encoded by the *DWF7* gene (also known as *STE1*), which is then converted to 24-methyleneCR (catalyzed by 7-dehydrocholesterol reductase encoded by the *DWF5* gene) (Choe, 2006; Ohnishi, 2018). Further stages of the C_28_-BR biosynthesis may proceed through two parallel pathways, called the late and early C-22 oxidation pathway. Reduction of 24-methyleneCR to campesterol initiates the late C-22 oxidation pathway and is catalyzed by the C-24(25)-sterol reductase in a two-step reaction in which 24-methyl-desmosterol is an intermediate (Dockter et al., 2014). The enzyme (also known as sterol side-chain reductase 1), which catalyzes the production of campesterol, is encoded by the *DWF1* gene. Campesterol is then transformed in the 5,4 isomerization reaction to (24*R*)-ergostan-4-en-3β-one. The latter is then converted through the DET2-mediated 5α-reduction to (24*R*)-5α-ergostan-3-one, which is transformed into campestanol (CN). In the parallel, early C-22 oxidation pathway, C-22α hydroxylation of 24-methyleneCR leads to the synthesis of 22-hydroxy-24-methyleneCR. The reaction of C-22α hydroxylation is catalyzed by the C-22α hydroxylase, which is encoded by the *DWF4* gene. The enzyme belongs to the P450 cytochrome family (Fujiyama et al., 2019). The next reactions are analogous to the late C-22 oxidation pathway and result in the synthesis of 22-hydroxy forms of the corresponding compounds. However, an essential difference between the C-22 oxidation sub-pathways is the synthesis of 6-deoxocathasterone (6-deoxoCT) from (22*S*,24*R*)-22-hydroxy-5α-ergostan-3-one, without synthesis of campestanol (CN) (CN-independent pathway of BRs biosynthesis) as a result of the early C-22 oxidation. On the other hand, in each stage of the late C-22 oxidation pathway, the compound can be hydroxylated by the C-22α hydroxylase into hydroxygenated forms of early C-22 pathway (Choe, 2004; Ohnishi, 2018). Moreover, biochemical changes of 22-hydroxymethyleneCR can lead to the synthesis of 6-deoxodolichosterone, which may be further converted into dolichosterone (DS), and dolicholide (DL), as well as to castasterone (CS) and BL (Roh et al., 2017).

Campestanol may be a substrate of the BR biosynthesis in a parallel manner, both in late C6 oxidation pathway (when hydroxylation of carbon atoms in the A-ring and both C-22 and C-23 positions of the side chain occurs before oxidation of C6) and early C-6 oxidation pathway (when hydroxylation takes place after oxidation of C6) (**Figure 1**; **Figure S1**) (Shimada et al., 2001). In *A. thaliana* both the early and late C6 oxidation pathways are functional (Fujioka et al., 2000); however, the late C6 oxidation pathway plays a prominent role during photomorphogenesis, whereas the parallel early C6 oxidation dominates during skotomorphogenesis (Noguchi et al., 2000). Generally, the late C6 pathway is more prominent in plants (e.g., in potato, it is the only type of the C_28_-BR biosynthesis). The late C6 pathway begins with hydroxylation of CN into the 6-deoxoCT by the 22α-hydroxylase. 6-deoxoCT may also be synthesized directly from 22-hydroxy5α-ergostan-3-one (the CN-independent pathway). Then, 6-deoxoCT is hydroxylated through the C-23 hydroxylase (encoded by the *CPD* gene) to the 6-deoxoteasterone, which is then C-3 oxidized into the 3-dehydro-6-deoxoteasterone (6-deoxo-3-DT) through the *CYP90D* C3-oxidase. In the next step, 6-deoxo-3-DT is converted to 6-deoxoTY. This reaction is catalyzed by the *D11* CYP724B1 enzyme. Then, 6-deoxoTY is hydroxylated to 6-deoxoCS by the 2α-hydroxylase encoded by the *DDWF1* gene. 6-deoxoCS is converted to castasterone (CS) (BR-6-oxidase1 and BR-6-oxidase2 catalyze the reaction). Then, CS is converted to BL *via* Baeyer-Villiger oxidation by the BR-6-oxidase2 (CYP85A2) (Choe, 2004; Choe, 2006; Vriet et al., 2013; Nakano and Asami, 2014; Ohnishi, 2018).

The early C6 oxidation pathway begins from hydroxylation of CN to 6α-hydroxyCN and its subsequent oxidation to 6-oxo-CN. The latter is transformed to CT by the 22α-hydroxylase. Cathasterone (CT) is converted in the consecutive reactions to teasterone (TE), 3-dehydroteasterone (3-DT), typhasterol (TY), CS, and BL, respectively (Shimada et al., 2001; Fujioka et al., 2002; Kwon and Choe, 2005; Ohnishi et al., 2006a; Ohnishi et al., 2006b; Lee et al., 2010; Lee et al., 2011; Zhao and Li, 2012; Chung and Choe, 2013; Joo et al., 2015; Kim et al., 2018; Ohnishi, 2018; Roh et al., 2020).

### Biosynthesis of C_29_-BRs

The C_29_-BR biosynthesis is the least known and described route of the BR biosynthesis (**Figure 1**; **Figure S1**). In this pathway 24-methylenelophenol is converted by sterol methyltransferase 2 (SMT2) into 24-ethylidenelophenol that is transformed into avenasterol by the sterol methyl oxidase 2. Then, isofucosterol and β-sitosterol are produced from avenasterol in a series of reactions catalyzed by the DWF7, DWF5, and SSR1 (DWF1) enzymes. β-sitosterol, as a precursor of the C_29_-BRs, is hydroxylated into 6-deoxo-28-homoTY and oxygenated into 28-homoTY by the *CYP724B2* and *CYP90B3* C-22 hydroxylase, respectively (Ohnishi et al., 2006a). 28-homoTY can be also formed from 28-homoTE, but intermediates of this reaction have not been identified yet. 28-homoTY is converted to 28-homoCS and 28-homoBL *via* the CYP85A1/A2 oxidases. The recent report suggests the way of 28-homodolicholide and CS synthesis from isofucosterol *via* 22-hydroxyisofucosterol, 6-deoxo-28-homoDS, and 28-homoDS, respectively. Moreover, CS can be converted from β-sitosterol, through the 22-homositosterol, 6-deoxohomositosterol, and 28-homoCS. It was found that 28-homoTE, 28-homoTY and 28-homoCS can be converted into 26-nor-28-homoTE, 26-nor-28-homoTY, and 26-nor-28-homoCS, respectively. C-26 demethylation might also serve to a deactivation of the C_29_-BRs (Lee et al., 2011; Joo et al., 2015; Roh et al., 2017; Kim et al., 2018).

### Links Between C_27_-C_28_ and C_28_-C_29_ Pathways

The C_27_-BRs biosynthetic pathway links with the C_28_ pathway through the following reactions: 28-norTE → TE, 28-nor-3-DT → 3-DT, 28-norTY → TY, and 28-norCS → CS. On the other hand, C_29_-BRs conversion to C_28_-BRs occurs through the following reactions: 28-homoTE → TE, 28-homoTY → TY, 28-homoCS → CS, 28-homoDS → CS, and 28-homoDS → DS → CS. Therefore, it is suggested that the biosynthetic connection of C_27_- and C_29_-BRs with C_28_-BRs occurs mainly between the end products of the pathways. Five pathways are biosynthetically connected to produce CS, an active BR, in plants (**Figure 1**; **Figure S1**). Direct substrates to synthesize CS are: 28-norCS, DS, 6-deoxoCS by 6α-hydroxyCS, 28-homoDS, and 28-homoCS. Thus, it is most conceivable that all the biosynthetic pathways of BRs in plants are funneled into CS to carry out the relevant biological activities. It is known that the early C-6 oxidation pathways of C_27_-, C_28_-, and C_29_-BRs are commonly interrupted in plant tissues (Fujita et al., 2006; Joo et al., 2012; Joo et al., 2015; Kim et al., 2018). A recent study of barley (*Hordeum vulgare*) BR mutants indicated that the accumulation of 28-homoCS is inversely correlated with the accumulation of CS: mutants deficient in the biosynthesis of CS accumulate the highest concentrations of 28-homoCS, on the other hand, the BR-insensitive line, in which the highest concentration of CS was observed, accumulates the lowest concentration of 28-homoCS (Gruszka et al., 2016).

## Inhibitors of BR Biosynthesis

**Figure 2:**
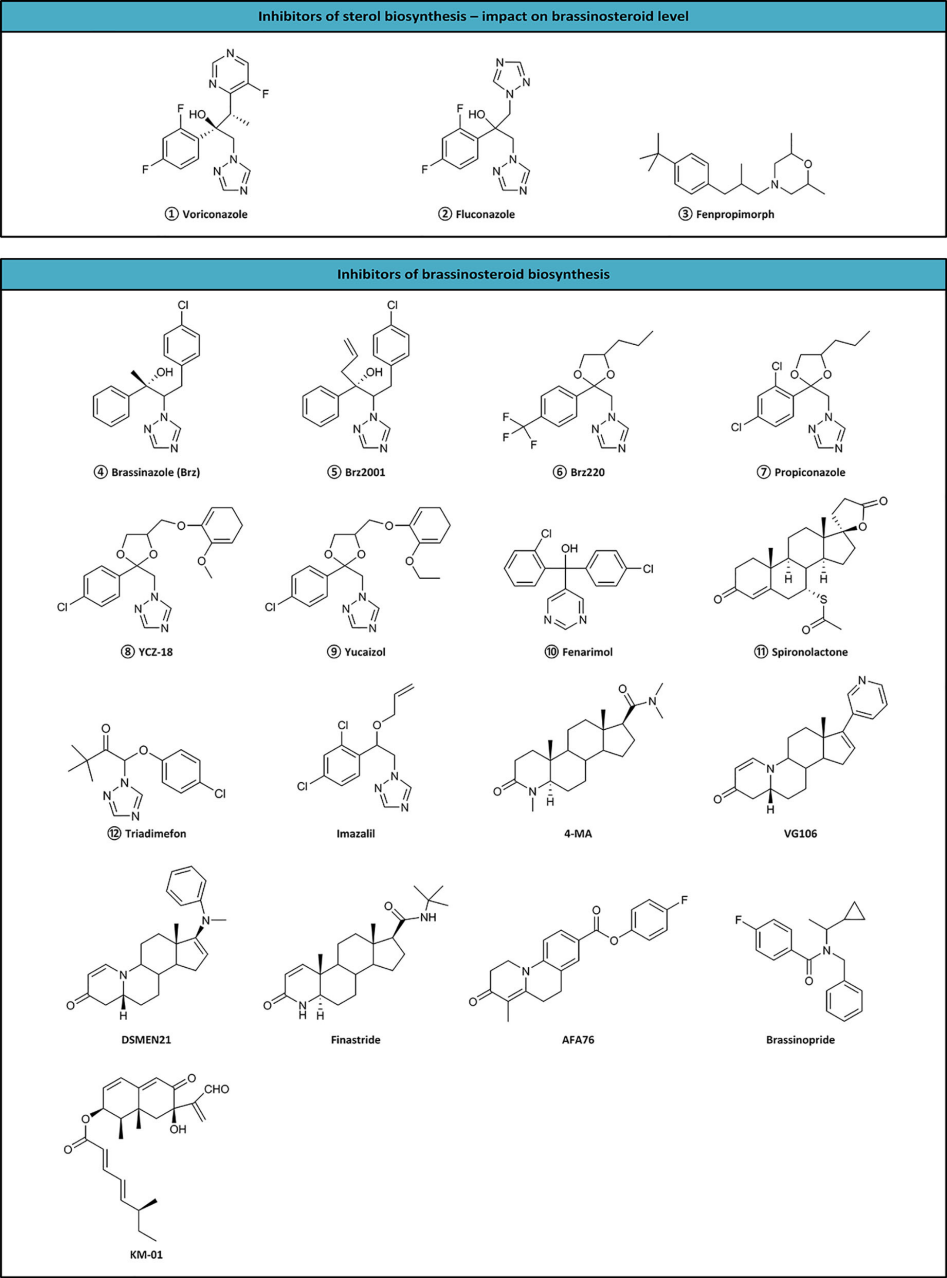
Inhibitors of sterol and brassinosteroid biosynthesis. Numbered inhibitors have a known site of action presented in **Figure 1**.

Inhibitors are tools useful not only for investigating biosynthetic pathways, but also for manipulating the BR level in crop plants. Till now, 17 inhibitors (KM-01, brassinozole (Brz), Brz2001, Brz220, propiconazole, YCZ-18, yucaizol, fenarimol, spironolactone, triadimefon, imazalil, 4-MA, VG106, DSMEM21, finastride, AFA76, and brassinopride) have been discovered (**Figure 2**), however, the site of action of only nine compounds is known. The sites of action of inhibitors are as follows:

- campestanol → 6-deoxoCT for brassinazole, Brz2001, Brz220, triadimefon, and spironolactone;
- 6-deoxoCT → 6-deoxoTE for brassinazole, Brz2001, Brz220, propiconazole, and fenarimol;
- 6-deoxoTE → 6-deoxo-3DT for YCZ-18, yucaizol, propiconazole, and fenarimol;
- 6-oxocampestanol → CT for brassinazole, Brz2001, Brz220, and triadimefon;
- CT → TE for brassinazole, Brz2001, Brz220, propiconazole, and fenarimol;
- TE → 3DT for YCZ-18, yucaizol, propiconazole, and fenarimol (Rozhon et al., 2019) (**Figure 1**; **Figure S1**).

The first reported BR inhibitor, i.e., KM-01 was isolated from a microbial culture medium. It inhibited BR activity in a rice lamina inclination test. Despite the unclear site of action, KM-01 exhibits highly potent activity (Kim et al., 1994; Kim et al., 1995; Kim et al., 1998). However, brassinazole (Brz) represents the first specific BR biosynthesis inhibitor, which blocks the conversion of campestanol to 6-deoxoCT, 6-deoxoCT to 6-deoxoTE, 6-oxocampestanol to CT, and CT to TE in the BR biosynthetic pathways. Brz2001 is a modified form of Brz containing an allyl moiety instead of the methyl group. Both inhibitors block the same reactions (Asami and Yoshida, 1999; Asami et al., 2000; Asami et al., 2001; Asami et al., 2003b). Brz and Brz2001 can induce morphological changes, including dwarfism, altered leaf color, and curling in de-etiolated barley (Sekimata et al., 2001). Brz decreased the level of BRs in the barley leaves, but not in roots. The inhibition effect of Brz on plant growth is reversed by exogenous BR (Bajguz and Asami, 2004; Bajguz and Asami, 2005; Bajguz et al., 2019).

Propiconazole, a triazole compound, also affects similar to Brz (Hartwig et al., 2012). Another triazole-type BR biosynthesis inhibitors, YCZ-18, and yucaizol, bind to the CYP90D1 enzyme and inhibit the BR-induced cell elongation. However, only BL negates the inhibitory effect of YCZ-18 or yucaizol. Therefore, it was suggested that they function differently from Brz (Oh et al., 2015a; Oh et al., 2015b). Fenarimol is known for inhibiting cytochrome P450 monoxides involved in 14α-demethylation during the biosynthesis of ergosterol. Simultaneously, it inhibits the conversion of CT to TE, and evokes the phenotype of BR-deficient mutants with short hypocotyls, de-etiolate dark-grown seedlings, and dark green downward curled leaves of light-grown *A. thaliana* (Wang et al., 2001; Oh et al., 2015a). Plants treated with triadimefon show reduced elongation of stems and petioles, dark green and thicker leaves, delayed senescence, and increased expression levels of the *CPD* gene. The phenotypes could be recovered with CT, TE, TY, CS, and BL (Asami et al., 2003a). On the other hand, imazalil causes severe hypocotyl shortening in *A. thaliana*, which could be reversed by the application of 24-epibrassinolide (Werbrouck et al., 2003). Seedlings of *A. thaliana* treated with spironolactone showed dark, downward curled leaves, and shortened hypocotyls, which could be reversed by BL application (Asami et al., 2004). Although *A. thaliana* mutants viz. *cpd*, *det2-1*, or *cbb1* treated with brassinopride enhanced apical hook formation, the normal phenotype was recovered by BL (Gendron et al., 2008). Voriconazole, fluconazole, and fenproprimorph (**Figure 2**) inhibit cycloeucalenol-obtusifoliol isomerase and have a reductive impact on BRs level (Rozhon et al., 2013; Rozhon et al., 2019).

## Author Contributions

AB and MC prepared a draft of figures and text. AB and DG corrected and finalized the review.

## Conflict of Interest

The authors declare that the research was conducted in the absence of any commercial or financial relationships that could be construed as a potential conflict of interest.

## Supplementary Material

Supplementary Figure 1 Multistep reactions of brassinosteroids biosynthesis and their sterol biosynthetic precursors.
