# Supplementary figures and images for: Comprehensive Overview of the Brassinosteroid Biosynthesis Pathways: Substrates, Products, Inhibitors, and Connections
Source: Front Plant Sci. 2020 Jul 7;11:1034. doi: 10.3389/fpls.2020.01034 (PMC7358554; doi:10.3389/fpls.2020.01034)

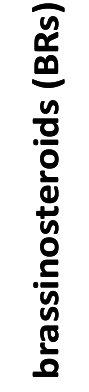

Supplement: Supplementary Figure 1 — Multistep reactions of brassinosteroids biosynthesis and their sterol biosynthetic precursors. [file Image_1.pdf]
